# Pancreatic Neuroendocrine Neoplasms: Basic Biology, Current Treatment Strategies and Prospects for the Future

**DOI:** 10.3390/ijms18010143

**Published:** 2017-01-13

**Authors:** Akihiro Ohmoto, Hirofumi Rokutan, Shinichi Yachida

**Affiliations:** Division of Cancer Genomics, National Cancer Center Research Institute, Tokyo 1040045, Japan; hrokutan@ncc.go.jp (H.R.); syachida@ncc.go.jp (S.Y.)

**Keywords:** pNENs, 2010 WHO classification, Ki-67 index, mitotic count, pNEC, tumor differentiation, whole-exome sequence data, everolimus, sunitinib, platinum regimen

## Abstract

Pancreatic neuroendocrine neoplasms (pNENs) are rare tumors accounting for only 1%–2% of all pancreatic tumors. pNENs are pathologically heterogeneous and are categorized into three groups (neuroendocrine tumor: NET G1, NET G2; and neuroendocrine carcinoma: NEC) on the basis of the Ki-67 proliferation index and the mitotic count according to the 2010 World Health Organization (WHO) classification of gastroenteropancreatic NENs. NEC in this classification includes both histologically well-differentiated and poorly differentiated subtypes, and modification of the WHO 2010 classification is under discussion based on genetic and clinical data. Genomic analysis has revealed NETs G1/G2 have genetic alterations in chromatin remodeling genes such as *MEN1*, *DAXX* and *ATRX*, whereas NECs have an inactivation of *TP53* and *RB1*, and these data suggest that different treatment approaches would be required for NET G1/G2 and NEC. While there are promising molecular targeted drugs, such as everolimus or sunitinib, for advanced NET G1/G2, treatment stratification based on appropriate predictive and prognostic biomarkers is becoming an important issue. The clinical outcome of NEC is still dismal, and a more detailed understanding of the genetic background together with preclinical studies to develop new agents, including those already under investigation for small cell lung cancer (SCLC), will be needed to improve the prognosis.

## 1. Introduction

Neuroendocrine neoplasms are derived from neuroendocrine cells throughout the human body, and the gastroenteropancreatic tract and lung are two main sites of this disease [1]. Pancreatic neuroendocrine neoplasms (pNENs) are a subtype of gastroenteropancreatic neuroendocrine neoplasms (GEP-NENs), and are distinguished from carcinoids according to the primary sites [2]. While pNENs are rare tumors accounting for only 1%–2% of all pancreatic malignancies, the incidence has increased substantially in the last four decades (from 1.09 to 5.25 per 100,000 individuals between 1973 and 2004), although improvement of diagnostic imaging may be the major reason for the apparent increase [1,3,4]. The newly published 2010 World Health Organization (WHO) classification of GEP-NENs and recent genomic data have greatly impacted the clinical management of this disease [5]. The 2010 WHO classification categorizes them into neuroendocrine tumor grade 1 (NET G1), neuroendocrine tumor grade 2 (NET G2) and neuroendocrine carcinoma (NEC) on the basis of the Ki-67 proliferation index and the mitotic count. Although this classification system is simple and practical, it has been criticized on the basis that it does not reflect real disease status. While notable progress in basic research, including genomic analysis, has led to improved clinical outcomes for NET G1/G2, treatment stratification is becoming an important issue. In regard to NEC, the median overall survival in patients with metastatic disease is generally less than one year [6,7], and new agents are urgently required for this dismal disease. Here, we review the basic biology and current treatment approaches for pNENs, and discuss prospects for effective treatment.

## 2. Histologic Classification

Histopathological classification of pNENs is performed according to the WHO classification of GEP-NENs, as revised in 2010 [5]. This classification system is a modification of a consensus proposal by the European Neuroendocrine Tumor Society [8]. The previous classification published in 2000 categorized GEP-NENs into three groups (well-differentiated endocrine tumor, well-differentiated endocrine carcinoma, poorly differentiated endocrine carcinoma/small cell carcinoma) [9], whereas the 2010 classification categorizes them into NET G1, NET G2 and NEC on the basis of the Ki-67 proliferation index and mitotic count [5]. A mitotic count of <2 per 10 high-power fields (hpf) and/or a Ki-67 index <3% corresponds to NET G1, a mitotic count of 2–20/10 hpf and/or a Ki-67 index of 3%–20% to NET G2, and a mitotic count of >20/10 hpf and/or a Ki-67 index >20% to NEC (Table 1). The mitotic count and Ki-67 index are parameters expressing the proliferation rate [2,10,11], and the higher of the two is adopted for categorization. Cytopathological features of NET G1/G2 are low mitotic rate, small to medium-sized ovoid nuclei, minimal pleomorphism, and lack of extensive necrosis, whereas those of NEC are high mitotic rate, high-grade cytological atypia, apparent pleomorphism, and extensive necrosis. Discordance between the mitotic count and Ki-67 index is sometimes observed in practical settings, and the clinical meaning of this phenomenon is under discussion. In a study of 285 patients with metastatic NENs, discordance between the above two parameters was detected in 44% of pNENs and 38% of midgut NENs patients [12]. Other studies found that patients with discordant pNENs had poorer overall survival (OS) than those with concordant neoplasms [13,14]. According to the 2010 classification, both small cell carcinoma and large cell neuroendocrine carcinoma (LCNEC) correspond to NEC. Except for one epidemiological study in the Netherlands, previous retrospective studies have found no significant difference of OS between these two subtypes, and uniform treatment approaches for small cell carcinoma and LCNEC might be reasonable on the basis of current data [7,15,16]. The North America Neuroendocrine Tumor Society (NANETS) guidelines for NENs also recommend the same treatment approaches for the two [17].

**Figure 1:**
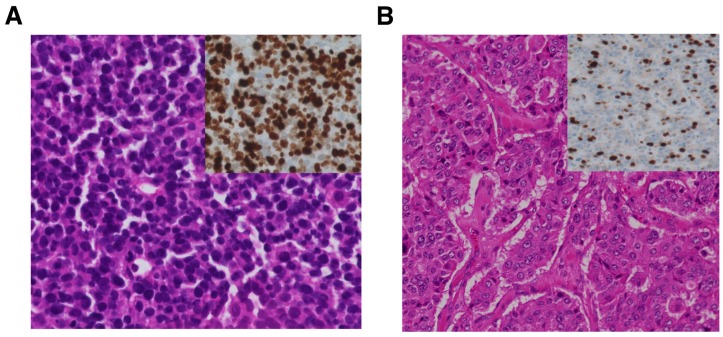
Histopathological features of poorly differentiated small cell carcinoma and well-differentiated NET G3. (**A**) In small cell carcinoma, tumor cells with a high nuclear cytoplasmic ratio and small-sized ovoid nuclei grow in a solid pattern. Immunohistochemical analysis shows extremely high Ki-67 labeling index values (hematoxylin and eosin, and Ki-67 immunohistochemical staining, original magnifications ×400). Ki-67 immunohistochemical staining is shown in the upper right corner; (**B**) in NET G3, tumor cells with a moderate amount of cytoplasm and a low mitotic rate show a trabecular and glandular growth pattern. The Ki-67 labeling index is over 20%, but is not as high as that of small cell carcinoma (hematoxylin and eosin, and Ki-67 immunohistochemical staining, original magnifications ×200). Ki-67 immunohistochemical staining is shown in the upper right corner.

This classification system is pathologically simple and very useful to standardize diagnosis and treatment procedures. However, one criticism is that tumor differentiation is not considered, and both well-differentiated and poorly differentiated tumors are included in NEC [15,18,19]. In other words, poorly differentiated small cell carcinoma or LCNEC meet the threshold for NEC, but well-differentiated neoplasms with a high Ki-67 index are also classified into NEC. The mitotic rate and Ki-67 index are higher in small cell carcinoma or LCNEC than in well-differentiated tumors. Therefore, some researchers have recently designated the latter as NET G3 to distinguish it from small cell carcinoma or LCNEC [20,21]. The conceptual diagram of NET G3 was shown in a review by Milione et al. [22]. Histopathological features of small cell neuroendocrine carcinoma and NET G3 are shown in Figure 1. Previous studies showed that disease-specific survival was significantly better in the well-differentiated subtype than in the poorly differentiated subtype [14,19,23,24], which suggests that the biological behaviors of the two are different. Recently, Milione et al. categorized 136 patients with NEC based on the 2010 classification into three groups (type A: well-differentiated and Ki-67 index 20%–55%; type B: poorly differentiated and Ki-67 index 20%–55%; type C: poorly differentiated and Ki-67 index ≥55%), and compared OS between these three groups. As a result, type A had the best OS out of the three, and OS in type C was especially poor compared to that in type B, which confirmed that the Ki-67 index as well as tumor differentiation was a independent prognostic factor for survival [25]. Tang et al. pointed out the existence of a high-grade component in well-differentiated NENs, and NET G1/G2 and NET G3 are speculated to be consecutive entities [24]. Morphological findings are the gold standard fundamental to the diagnosis of NET G3, whereas we will focus on how to add genetic data, as shown in the next section, into the current classification system.

## 3. Genetic Characterisics

Recent large-scale genomic data has improved our understanding of pNENs and provided critical information for the development of new agents in this field. Key differences of genetic background between poorly differentiated NEC and other types of pNENs have gradually been revealed. Therefore, in this section, we review the genetic characteristics of pNENs, focusing on the distinction of the poorly differentiated subtype from the well-differentiated subtype.

Whole-exome sequence data reported by Jiao et al. in 2011 was a milestone in understanding the molecular biology of well-differentiated pNENs [26]. They analyzed 10 pNENs by whole-exome sequencing and also conducted targeted sequencing of 58 additional cases for validation (Table 2).

First, somatic inactivating mutations in *MEN1* were detected in 44% of the cases [26]. *MEN1* is a tumor suppressor gene related to multiple endocrine neoplasia type 1 syndrome, and a germline inactivating mutation in one allele of this gene and a second somatic mutation in the other allele interact at the onset of this syndrome through the loss of heterozygosity or intragenic mutation [27]. The protein menin produced by the *MEN1* gene is one component of the MLL/SET1-like histone methyltransferase complex and regulates gene transcription by coordinating chromatin remodeling. Although somatic mutations of *MEN1* were first discovered in familial pNENs, they are observed in sporadic cases as well as in hereditary ones, and genetic analysis of 100 sporadic pNENs showed that 25% had somatic mutations in *MEN1* [28].

Second, somatic inactivating mutations in *ATRX* (α thalassemia/mental retardation syndrome X-linked) and *DAXX* (death-domain associated protein) were detected in 18% and 25% of the cases, respectively [26]. These two mutations do not occur concurrently in the same tumor, which suggests that the encoded proteins function in the same pathway [27]. The proteins encoded by *ATRX* and *DAXX* interact with one another, and are related to chromatin remodeling at telomeres. There is a strong correlation between the inactivation of *ATRX* or *DAXX* and the telomerase-independent telomere maintenance mechanism termed alternative lengthening of telomeres (ALT), and loss of *ATRX* is recognized as a hallmark of ALT cell lines [29]. Although the ALT phenotype is common among certain types of tumors such as sarcomas and central nervous system tumors, the prevalence of the ALT phenotype was only 4% in various other tumor types, so that a high mutation rate (43%) of *ATRX* or *DAXX* appears to be a characteristic genomic feature in pNENs [30]. Jiao et al. claimed that these mutations were associated with a better prognosis, but Singhi et al. performed telomere-specific FISH and *DAXX*/*ATRX* immunohistochemistry of 373 pNENs patients and concluded that ALT and *DAXX*/*ATRX* loss were associated with a worse prognosis [31]. Therefore, the clinical impacts of mutations in these two genes remain controversial. *ATRX* and *DAXX* had not previously been associated with cancer, so these findings have attracted much attention.

Third, somatic mutations in genes associated with the mammalian target of rapamycin (mTOR) pathway were detected in 18% of patients [26]. Specifically, the prevalence of mutations was 7% for *PTEN*, 9% for *TSC2* and 1% for *PIK3CA*. Mutations in *PTEN* and *TSC2* are inactivating mutations, whereas the mutation in *PIK3CA* is regarded as oncogenic, involving a hotspot for activation of the kinase domain of the encoded protein [27,32]. As mentioned in the next section, mTOR inhibitors are in clinical use, and the detection of mutations in *PTEN*, *TSC2* and *PIK3CA* is very important in considering specific treatment approaches for pNENs.

Raj et al. also presented whole-exome sequence data for 44 patients with well-differentiated pNENs at the 2016 American Society of Clinical Oncology (ASCO) annual meeting [33]. As shown in Table 2, their data were generally in line with the sequence data by Jiao et al., although *SETD2* mutations were newly detected in 21% of the cases. The protein encoded by *SETD2* is related to chromatin remodeling, and inactivating mutations of this gene are reported in clear cell renal cell carcinoma [34].

In contrast, no whole-exome sequence data is available for poorly differentiated NEC, and the precise genetic background of NEC is unknown. Reasons for this include the rarity of this disease and the paucity of resectable cases (and hence surgical specimens). Yachida et al. performed immunohistochemical analysis for 19 poorly differentiated pancreatic NEC (pNEC) cases, and found abnormal immmunolabeling of the p53 protein and Rb protein in 95% and 74% of the cases, respectively, and 74% of the cases overexpressed Bcl-2 protein [35]. In Sanger sequencing, 57% of the cases had inactivating mutations of the *TP53* gene and 71% had inactivating mutations of the *RB1* gene (Table 3). Hijioka et al. also reported that immunopositivity of the Rb protein was detected in only 14% of poorly differentiated NEC [36]. Large-scale whole-genome sequence data for small cell lung carcinoma (SCLC) revealed inactivation of *TP53* and *RB1* in 100% and 93% of the cases, which suggests a similarity of the genomic background between SCLC and pNEC [37]. At the 2016 ASCO annual meeting, Bergsland et al. presented targeted-sequencing data (192 cancer-related genes) for 593 SCLC cases and 274 GEP-NEC cases, including 123 pNEC cases [38]. The prevalence of mutations in pNEC was 18% for *TP53*, 10% for *RB1*, 33% for *MEN1* and 20% for *DAXX*, indicating that mutations in *TP53* and *RB1* are less common in pNEC than in SCLC, colon NEC and other types of gastrointestinal (GI)-NECs, whereas mutations in *MEN1* and *DAXX* are more common in pNEC (Table 3). This is the first study to identify different mutation patterns for each primary site within the GEP system and between GEP-NEC and SCLC. In addition, another genetic feature of pNEC is the low prevalence of *KRAS* mutations, as shown in Table 3. This is opposite to pancreatic ductal adenocarcinomas (PDAC), which almost all have *KRAS* mutations, indicating that pNEC and PDAC are genetically different entities [39].

Genomic information is expected to provide a rationale for the modification of the current WHO classification system. Tang et al. proposed the new diagnostic algorithm secondarily adding genetic information into conventional morphologic diagnosis for pancreatic NEC [23]. Here, they categorized cases with loss of *DAXX* and *ATRX* expression into well-differentiated NEC (NET G3), whereas cases with loss of Rb and abnormal p53 expression were categorized into poorly differentiated NEC, improving the accuracy of diagnosis especially for unclassifiable cases by morphologic diagnosis. Although we currently obtain little sequence data for NET G3 and the situation of genetic information is supplementary, molecular-based definitions would refine classical histological classifications in the near future.

**Figure 2:**
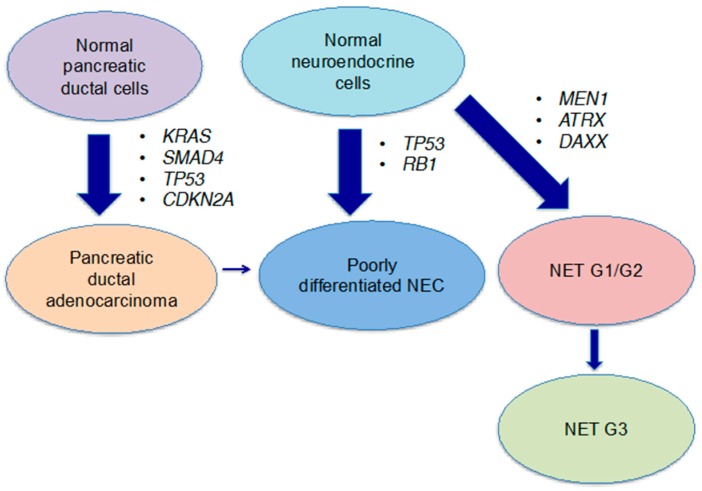
A conceptual diagram of the onset of pNEC. Poorly differentiated NEC is considered to be derived mainly from normal neuroendocrine cells through inactivating mutations of *TP53* and *RB1*. In addition, some NET G1/G2 cases histologically progress to well-differentiated NET G3, and few cases of NEC are speculated to derive from PDAC cells.

Based on these genomic data, the molecular mechanism in poorly differentiated pNEC is speculated to be as follows: most pNEC cases derive from normal neuroendocrine cells without going through pNET G1/G2 or PDAC, and inactivating mutations of *TP53* and *RB1* directly lead to the onset of this disease (Figure 2). A case report, however, suggested the possibility of pNEC with activating the *KRAS* mutation and aberrant expressions of *TP53* and *SMAD4*, and Hijioka et al. found that the *KRAS* mutation was present in over 80% of cases of poorly differentiated NEC [36,40]. These findings suggest that a part of pNEC might be derived from PDAC cells.

## 4. Current Treatment Strategies

### 4.1. Well-Differentiated pNENs (NET G1/G2)

Surgical resection with regional lymph node dissection is the only curative treatment option, and is recommended to all patients with early-stage well-differentiated pNENs [41,42,43]. Although cancer patients with metastatic diseases are generally unsuitable for surgical resection in the field of oncology, partial hepatectomy is often performed in pNENs patients with liver metastases, depending upon the number, size and location of the lesions, the extent of the primary tumor and the patient’s performance status [43]. The rationale is provided by studies showing longer survival after resection of liver metastases, and the clinical effectiveness of liver resection can be partly explained by intrinsic slow progression of well-differentiated pNENs [44,45]. When liver lesions are not resectable, radiofrequency ablation and transarterial chemoembolization are frequently employed as palliative approaches [46].

For patients with advanced disease, systemic therapies form the mainstay. However, advanced cases are incurable, and observation is an option in patients with low tumor volume [17]. Somatostatin analogues are effective for controlling the hormone-excess state in functional pNENs through effectively reducing the release of peptides and neuroamines. Moreover, these drugs also have an antiproliferative tumor effect in functional and non-functional pNENs [2,47]. NENs overexpress any of the five subtypes of somatostatin receptor (SSR) in over 70% of cases, and the functions of SSR are type-specific [48]: mainly inhibition of hormone secretion for SSR types 2, 5; cell cycle arrest for SSR types 1, 2, 4, 5; and apoptosis for SSR type 3. Somatostatin analogues control SSR-positive tumors through these molecular pathways, and the activity of each analogue is determined by its affinity for SSR [43,48,49]. In the phase III Controlled Study of Lanreotide Antiproliferative Response in Neuroendocrine Tumors (CLARINET) trial, 204 patients with nonfunctional grade 1–2 GEP-NENs including pNENs were randomized to receive long-acting somatostatin analogue lanreotide or placebo [50]. Progression-free survival (PFS) was significantly better with lanreotide (median PFS, not reached vs. 18 months; hazard ratio (HR), 0.47; 95% confidence interval (CI), 0.30–0.73; *p* < 0.001). The Phase III Placebo-Controlled Prospective Randomized Study on the Antiproliferative Efficacy of Octreotide LAR in Patients with Metastatic Neuroendocrine Midgut Tumors (PROMID) trial for well-differentiated midgut NENs showed that another somatostatin analogue, octreotide long-acting release (LAR), extended PFS compared with placebo (median PFS, 14.3 vs. six months; HR, 0.34; 95% CI, 0.20–0.59; *p* < 0.001) [51]. Low response rates to somatostatin analogues have been noted in previous studies, suggesting that the clinical effects in most cases are caused by maintaining stable disease and/or by slowing disease progression. Therefore, aggressive disease may not be susceptible to this approach, and patients with a low and slowly progressive tumor burden are best suited for this therapy [52].

Peptide receptor radiotherapy (PRRT) is a novel treatment modality for advanced well-differentiated pNENs, and has developed in conjunction with imaging of radiolabeled SSR. The strategy of coupling an isotope such as ^177^Lu or ^90^Y with a somatostatin analogue through a linker has been used to develop therapeutic agents [49]. PRRT is very effective against SSR-positive tumors, but is inapplicable to tumors not expressing SSR [53]. Further, ^90^Y-DOTA-Tyr^3^-octrotide is a representative agent in this field, and a phase II trial for 1109 patients with metastatic NENs showed a 34% response rate (RR) [54]. At the 2016 Gastrointestinal Cancers Symposium, Strosberg et al. presented clinical results from the phase III NETTER-1 trial for midgut NENs [55]. In this trial, 230 patients with metastatic grade 1–2 midgut NENs were randomized to ^177^Lu-DOTA-Tyr^3^-octreotate and octreotide LAR, and the former extended PFS significantly (median PFS, not reached vs. 8.4 months; HR, 0.21; 95% CI, 0.13–0.34; *p* < 0.0001). Although this trial provided impressive data that changed clinical practice, it was performed for midgut NENs, and effects on NENs derived from other organs are unproven.

Conventional cytotoxic agents have been used to treat progressive metastatic diseases for over 50 years. Streptozocin is the most widely used alkylating agent for NENs in many countries, and is usually combined with another cytotoxic agent such as fluorouracil or doxorubicin [43]. In a randomized trial, the combination of streptozocin and doxorubicin demonstrated improved activity compared with that of streptozocin and fluorouracil [56]. However, this result was not reproduced in similar studies conducted later, and the best streptozocin regimen remains unknown. Recently, the combination of another alkylating agent, temozolamide, with capecitabine showed an impressive RR of 70% [57]. The positioning of these regimens in the clinical setting is still under discussion. However, there have been no cytotoxic agents demonstrating longer OS or PFS for pNENs, and in any case such agents are generally not a first choice because of their severe toxicities [43]. On the other hand, the RR is higher than those of other treatment options, and chemotherapy is an effective treatment for symptomatic and clinically aggressive cases [49,58].

**Figure 3:**
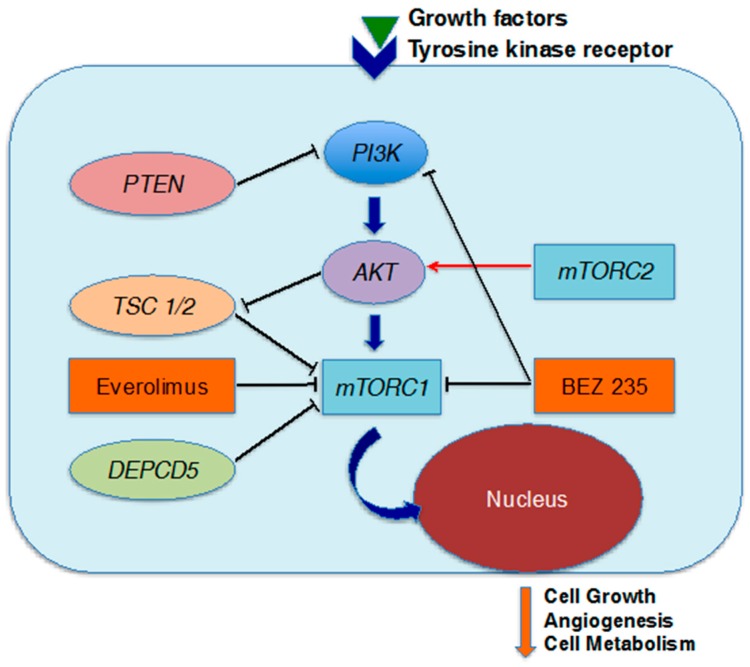
The mTOR signaling pathway as a target of molecular therapies. At the onset of pNENs, the upregulation of mTOR complex 1 (mTORC1) leads to cell growth, angiogenesis and cell metabolism. Everolimus suppresses mTORC1, and BEZ235 concurrently suppresses PI3K and mTORC1. Red arrows represent activation and black bars inhibition in each cascade.

Molecular targeted drugs are considered one of the most interesting potential therapies for advanced pNENs as a result of notable progress in basic research. At this stage, the mammalian target of rapamycin (mTOR) inhibitors and tyrosine kinase inhibitors appear to have great potential. Whole-exome sequence data revealed somatic mutations in genes associated with the mTOR pathway in 18% of cases, and the mTOR pathway is an important cascade regulating cell growth/proliferation, angiogenesis and cell metabolism at the onset of pNENs [26,27]. The mTOR inhibitor everolimus suppresses multiprotein complexes termed mTORC1 and inhibits downstream signaling [59] (Figure 3). In the phase III RAD001 in Advanced Neuroendocrine Tumors (RADIANT)-3 trial, 410 patients with advanced low- to intermediate-grade pNET with progression within the previous 12 months were randomized to receive everolimus or placebo, and everolimus markedly extended the PFS (median PFS, 11.0 months vs. 4.6 months; HR, 0.35; 95% CI, 0.27–0.45; *p* < 0.001) [60]. Thereafter, in the phase III RADIANT-4 trial, 302 patients with advanced well-differentiated NET of the lung and gastrointestinal tract were randomized to receive everolimus or placebo; in this trial, PFS as a primary endpoint was significantly better with everolimus (HR, 0.48; 95% CI, 0.35–0.67; *p* < 0.00001), confirming the clinical effectiveness of everolimus for NENs derived from other organs, as well as pNENs [61]. However, therapeutic resistance is often encountered, and various mechanisms may be involved. For example, inhibition of mTORC1 causes upregulation of PIK3 and AKT by relieving negative feedback, mTORC2-mediated AKT activation, and activation of other receptor tyrosine kinases [62,63,64,65]. Considering these mechanisms, the use of combinations of agents regulating different parts of the PI3K/AKT/mTOR pathway might be an effective strategy. BEZ 235 is a potent oral PI3K and mTOR inhibitor, and a randomized phase II trial of BEZ 235 in patients with advanced pNENs has been conducted [66] (Figure 3). This agent failed to provide a longer PFS for mTOR inhibitor–naïve patients in comparison with everolimus, but this approach warrants further investigation.

High vascular density is one of the characteristic features of well-differentiated NENs and is becoming a therapeutic target [67]. Sunitinib is a small-molecular, multi-target antiangiogenic tyrosine kinase inhibitor that blocks the vascular endothelial growth factor receptor (VEGFR) as well as platelet-derived growth factor receptor (PDGFR)β, c-KIT, FlT-3 and RET [60]. In a phase III trial, 171 patients with advanced well-differentiated pNET were randomized to receive sunitinib 37.5 mg daily or placebo, and sunitinib treatment resulted in a longer PFS (median PFS, 11.4 months vs. 5.5 months; HR, 0.42; 95% CI, 0.26–0.66; *p* < 0.001) [68]. However, therapeutic resistance to this agent appears to arise through the following mechanism: inhibition of the VEGF signaling pathway leads to hypoxic stress, which induces upregulation of transcription factors controlling the expression of multiple pro-angiogenic molecules [69]. Everolimus and sunitinib were approved in the United States and European countries for pNENs based on the above two randomized phase III studies. While these two agents extend PFS, there have been no direct comparative studies, and either of them can be used as a first choice for pNENs, taking into account the adverse events associated with each agent. The profiles of adverse events are different between the two agents: specific side effects of everolimus are pneumonitis and hyperglycemia, whereas those of sunitinib are hypertension and hand-foot syndrome [60,68]. Both agents have a modest RR (<10%), and delay of tumor growth is regarded as their main action. Concurrent inhibition of VEGF and the mTOR pathway is regarded as an effective strategy to overcome resistance. Combination therapy with mTOR inhibitor temsirolimus and VEGF-A antibody bevacizumab showed an RR of 41% [70]. A phase II study randomizing patients with metastatic pNENs to receive everolimus with or without bevacizumab showed a higher RR in the combination group (31% vs. 12%, *p* = 0.005) [71]. However, severe adverse events were also more common in the combination group, and less toxic treatments are needed.

The antiangiogenic tyrosine kinase inhibitor pazopanib is another promising agent. In a phase II trial, pNENs patients receiving pazopanib combined with depot octreotide had a median PFS of 14.4 months, which is comparable to that obtained with everolimus or sunitinib [72]. Thalidomide is an antiangiogenic and immunomodulating agent, and a phase II trial evaluated the clinical effect of combination therapy with temozolomide and thalidomide for patients with metastatic GEP-NENs, including pNENs and pheochromocytomas. The RR in the entire cohort was 25%, and reached 45% in pNENs [73]. Various other clinical trials of new agents for pNENs are also being conducted (Table 4) [74]. Although the arrival of new agents gives patients more therapeutic options, treatment stratification becomes increasingly important. In addition, the development of better predictive and prognostic biomarkers will be essential to identify the optimum agent for each patient [2,74].

### 4.2. Poorly Differentiated NEC

Pancreatic NEC is a rare tumor accounting for less than 2%–3% of all pancreatic NETs [5]. Contrary to the situation for NET G1/G2, the role of surgical resection for NEC is unclear, because many cases are unresectable and most of the resectable cases encounter recurrence or metastasis within one year [75]. Therefore, chemotherapy is the main therapeutic option for this disease. There is no established standard chemotherapy for GEP-NEC, and a platinum-based combination regimen is widely used as first-line chemotherapy on the basis of the treatment strategy for SCLC [17]. Among various platinum-based regimens, the cisplatin (CDDP) and etoposide (ETP) combination regimen (EP regimen) is the most widely used all over the world [76]. Sorbye et al. retrospectively analyzed 305 patients with advanced GEP-NEC [7]. Among them, 51% received an EP regimen, 27% a carboplatin (CBDCA) and ETP combination regimen and 11% a CBDCA, ETP and vincristine combination regimen. The median OS in all patients receiving chemotherapies was 11 months, and there was no significant difference among the platinum regimens. This study also revealed that patients with a Ki-67 index <55% had a lower RR and better survival, which suggests that NEC in the WHO classification might not be a single entity. Although the above study did not evaluate the differentiation status of each tumor specimen, a specific approach for well-differentiated NEC (NET G3) might be considered in the future. Yamaguchi et al. reviewed 258 patients with advanced GEP-NEC, and compared GI-NEC and hepato-biliary-pancreatic system (HBP) NEC [6]. A CDDP and irinotecan (CPT-11) combination regimen (IP regimen) is mainly employed for GI-NEC and an EP regimen for HBP-NEC; the RR and OS were better with the IP regimen than with the EP regimen (RR, 50% vs. 28%; OS, 13.0 vs. 7.3 months). However, these were retrospective analyses, and prospective studies are required for the comparison of clinical effects between regimens. At the same time, the above two studies showed that clinical practice is affected by regional factors (Western vs. East Asia), and survival data were dismal even in patients receiving platinum regimens. Based on meta-analysis and a phase III trial for SCLC, CBDCA and CPT-11 are practically recognized as equally effective compared to CDDP and ETP, respectively [77,78]. However, retrospective studies indicated that response to platinum regimens and survival are different between SCLC and extrapulmonary NEC, which suggests that extrapolation from SCLC data is not necessarily reliable [79,80,81]. The first randomized phase III trial between EP and IP regimens in GEP-NEC patients (JCOG 1213 trial) is currently being conducted by the Japan Clinical Oncology Group [82]. Another US intergroup randomized phase II trial for GEP-NEC comparing a temozolomide and capecitabine combination regimen with an EP regimen is underway (NCT 02595424). There are no established regimens for second-line treatment so far. NANETS guidelines recommend the same regimen as the first-line treatment for cases relapsing over six months after the termination of first-line therapy, and suggest a change to another regimen for cases relapsing within three to six months [17]. As for non-platinum regimens, retrospective studies demonstrated a good response and OS with a temozolomide-based combination regimen or the combination of 5-fluorouracil, leucovorin, and irinotecan (FOLFIRI) regimen [83,84]. Considering the high recurrence rate after surgery, adjuvant platinum-based chemotherapy for resectable cases is proposed, and NANETS guidelines recommend four to six postoperative cycles of chemotherapy [17]. In the future, the potential of adjuvant chemotherapy should be evaluated prospectively.

Although the introduction of innovative molecular targeted drugs has brought about significant advantages in treatments for various types of cancers, no new agents have yet been approved for pNEC. One reason for this may be our incomplete understanding of the genetic background in this disease. Although there has also been no breakthrough in the treatment of SCLC, which has a similar genetic background, some promising new agents have emerged in recent years. Immune checkpoint inhibitors are currently attracting great interest among oncology researchers and clinicians. In the CheckMate-032 trial randomizing pretreated SCLC patients to receive the combination of PD-1 inhibitor nivolumab and CTLA-4 inhibitor ipilimumab or nivolumab as a single agent, the RR was 21% in the combination group compared to 10% for nivolumab [85]. Another PD-1 inhibitor, pembrolizumab, showed an RR of 25% in the KEYNOTE-028 trial for SCLC patients [86]. Rovalpituzumab tesirine (Rova-T) is an antibody drug conjugate targeting delta-like protein 3 (DLL3), and a clinical trial for patients with recurrent or refractory SCLC showed an RR of 25% in all cases and 55% in DLL3-positive tumors [87]. Tarextumab, targeting NOTCH receptors, showed an RR of 84% in combination with the EP regimen for untreated advanced SCLC [88]. These new agents under investigation for SCLC might also be good candidates for GEP-NEC, and preclinical studies using GEP-NEC models might prove fruitful.

## 5. Conclusions and Future Directions

The new 2010 WHO classification of GEP-NENs is simple and practical, and has greatly contributed to the standardization of diagnosis and treatment procedures for pNENs. On the other hand, this system does not consider tumor differentiation, and there are calls for its modification. Recently published large-scale genomic data has improved our understanding of pNENs, and is beginning to uncover the differences in the genetic background between NET G1/G2 and NEC, as well as the genetic similarities between pNEC and SCLC. In terms of treatment, promising agents such as everolimus and sunitinib have emerged for NET G1/G2, and there is now a need for the development of adequate biomarkers to enable proper treatment stratification.

On the other hand, the prognosis of NEC is still dismal under standard platinum combination regimens, which strongly suggests the importance of further basic and translational research on this disease. Intergroup studies are essential to find efficient clinical treatments for rare tumors such as pNEC, and some agents under investigation for SCLC might be good candidates. Another point is that direct treatment approaches for currently known inactivating mutations of *TP53* and *RB1* are difficult, and detailed genetic analysis of pNEC to discover new oncogenes might provide a breakthrough for the conquest of this disease. Finally, as increasing amounts of medical data become available, we have to integrate and fit these clinical, pathological and genomic data into a clinical context.

## Figures and Tables

**Table 1:** WHO 2010 classification of GEP-NENs.

Classification	Mitotic Count (per 10 hpf)	Ki-67 Index (%)
NET G1	<2	<3
NET G2	2–20	3–20
NEC	>20	>20

WHO, World Health Organization; GEP, gastroenteropancreatic; NENs, neuroendocrine neoplasms; NET, neuroendocrine tumor; NEC, neuroendocrine carcinoma.

**Table 2:** Prevalence of common gene mutations for well-differentiated pNENs.

Gene	Jiao et al. [26]	Raj et al. [33]
*MEN1*	44%	61%
*ATRX*	18%	25%
*DAXX*	25%	41%
*PTEN*	7%	11%
*TSC1/TSC2*	9%	18%
*PIK3CA*	1%	NA
*ARID1A*	NA	14%
*SETD2*	NA	21%

pMENs, pancreatic neuroendocrine neoplasms; NA, not available.

**Table 3:** Prevalence of common gene mutations for pNEC by targeted-sequencing data.

Gene	Yachida et al. [35]	Bergsland et al. [38]
*TP53*	57%	18%
*RB1*	71%	10%
*CDKN2A*	0%	21%
*CDKN2B*	NA	16%
*KRAS*	29%	7%
*MEN1*	NA	33%
*DAXX*	NA	20%

pNEC, pancreatic neuroendocrine carcinoma; NA, not available.

**Table 4:** Clinical trials using new agents for pNENs.

Agent	Mechanism	Phase	Status
Romidepsin	HDAC inhibitor	II	Terminated
Motesanib + Octreotide	Multi-tyrosine kinase inhibitor	II	Completed
Ganitumab	Human anti–insulin-like growth factor receptor type I monoclonal antibody	II	Ongoing
MK-2206	AKT-inhibitor	II	Completed
Cabozantinib	Multi-tyrosine kinase inhibitor	II	Ongoing
X-82 + everolimus	VEGFR tyrosine Kinase Inhibitor	I/II	Ongoing
Endostatin + temozolomide/dacarbazine-based chemotherapy	The 20 kDa C-terminal fragment of collagen XVIII	II	Ongoing
Famitinib	Multi-tyrosine kinase inhibitor	II	Ongoing
Fosbretabulin	Microtubule destabilizing agent	II	Ongoing
Carfilzomib	Proteasome inhibitor	II	Ongoing
Ribociclib	CDK 4/6 inhibitor	II	Ongoing
Sulfatinib	Tyrosine kinase inhibitor of VEGFR 1, 2 and 3 and FGFR 1	III	Ongoing
Ibrutinib	Bruton’s tyrosine kinase inhibitor	II	Ongoing
Palbociclib	CDK 4/6 inhibitor	II	Ongoing

pNENs, pancreatic neuroendocrine neoplasms; HDAC, histone deacetylase; VEGFR, vascular endothelial growth factor receptor; CDK, cyclin-dependent kinase; FGFR, fibroblast growth factor receptor.
